# Necrotizing enterocolitis: perspectives from a population-based study

**DOI:** 10.1038/s41390-025-03884-7

**Published:** 2025-01-21

**Authors:** George S. Bethell, Nigel J. Hall

**Affiliations:** University Surgical Unit, Faculty of Medicine, University of Southampton, Southampton, UK

Invited commentary of *Kim, S. H., Son, J. & Park, H. K. Surgical Necrotizing Enterocolitis Risk Factors in Extremely Preterm Infants: A Korean Nationwide Cohort Study. Pediatr Res (2024)*.

In this current issue, Kim and colleagues report findings from a nationwide neonatal registry in the Republic of Korea with focus on Necrotizing Enterocolitis (NEC). Data over a 7 year period were interrogated to identify risk factors for medical NEC and surgical NEC amongst a cohort of over 5000 infants born at less than 28 weeks.^1^ Importantly, those who died of NEC prior to surgery were included in the surgical group. A relatively high incidence of NEC was observed (13.5%) when compared to a recent systematic review reporting incidence in identical populations of between 2 and 8.7%.^2^

Interestingly, the rate of surgical intervention in this cohort (68.6%) is higher than many previous studies and likely reflects the low gestational age of infants in this study. In an even younger population, a UK-based centre found that 55% of those born less than 24 weeks corrected gestational age (CGA) with NEC underwent surgical intervention.^3^ It is however possible that the current study missed some infants treated medically for NEC which might reflect why no risk factors for medical NEC were identified after adjusting for gestational age.^1^ Infants were only included if they were classified as modified Bell’s stage of two or more, hence pneumatosis intestinalis or portal venous gas on radiograph are required. These radiological findings can be difficult to detect and as many as 82% of infants born at less than 24 weeks CGA with NEC at laparotomy did not have pneumatosis on pre-operative imaging in a previous study.^3^ Defining NEC for research studies is challenging with many definitions existing and the difficulty of excluding similar, but separate conditions, such as focal intestinal perforation (FIP).^4,5^ This current study is strengthened by the exclusion of those with FIP.

The authors of the current manuscript have identified risk factors for surgical NEC which include being small for gestational age, hypotension (defined as requiring inotropes in the first week of life) and intraventricular haemorrhage (Papile grade II or higher) whilst complete administration of antenatal steroids appears to be protective.^1^ A recent meta-analysis also exploring this question included 18 studies and along with reduced gestational age found several other factors associated with surgical NEC including high C-reactive protein, thrombocytopaenia, high procalcitonin, low birthweight, leukocytopaenia, low plasma sodium and increased days since NEC onset.^6^ The clinical implications of these data are interesting to consider since due to study methodology these ‘risks factors’ are implied by association rather than causation. However, as the authors propose, the presence of any one of these factors may alert the clinician that a baby is at high risk of developing surgical NEC. One can imagine that subsequent practice may include providing interventions associated with reduced risk of NEC whilst simultaneously closely monitoring for NEC and if it were to develop, being alert to the presence of indications for surgery.

Moving beyond associations to developing a predictive model or identifying value cut-offs in variables that may accurately predict an event is a challenge addressed across all areas of medical research including NEC. In NEC there is growing interest in being able to distinguish infants with surgical NEC from infants with medical NEC early in their disease course. A recent systematic review found 114 reports of 63 unique tests to identify surgical NEC, with an exponential growth in publications in recent years.^7^ Many tests were derived in single centre datasets without internal validation. Hence, further work, externally validating existing methods is much awaited.

There were 47 infants (6.5%) in this study by Kim et al. who died of NEC prior to surgical intervention taking place.^1^ Battersby et al. reported data from a UK based neonatal dataset and found that this rate was higher (21%) with slightly different inclusion criteria which were infants <32 weeks CGA with severe NEC, defined as NEC confirmed at laparotomy or death attributed to NEC.^8^ These figures are a reminder that we urgently need to better understand which infants should be transferred to surgical neonatal units and which criteria indicate referral to the surgical team. To achieve this, we need to identify infants early in their disease course that are likely to deteriorate, or not respond to medical therapy. As such the variables identified by Kim and colleagues as being associated with surgical NEC could be considered for this purpose. However any changes to referral criteria must be carefully balanced against the risk of increased transfer rates of premature infants to surgical centres without evidence of benefit.

As with most registry-based studies investigators are limited to variables already collected and an understanding of population level data comes at the cost of missing some granularity surrounding modifiable risk factors for NEC. Despite extensive research efforts the only clear modifiable risk factor to prevent NEC is use of breast milk, rather than formula, which was found to half the risk of NEC on meta-analysis.^9^ There is growing interest in use of probiotics with good evidence to support their use in a number of studies.^10^ Further work in this area is underway to understand which probiotics are most effective and when these should be used.^11^ It would be useful to understand feeding strategies and probiotic use in this Korean cohort of infants.

In conclusion, Kim and colleagues have provided an important perspective at population level of NEC, reporting risk factors for medical and surgical disease similar to other population-based studies. These data highlight the importance of identifying modifiable risk factors, developing management strategies or therapies to address these, and earlier identification of disease progression, necessitating escalation of treatment and consideration for surgical intervention.
